# Supplementary material for: Differences in right-to-left vs left-to-right interventricular conduction times in patients indicated to cardiac resynchronization therapy
Source: PLoS One. 2020 Feb 19;15(2):e0228731. doi: 10.1371/journal.pone.0228731 (PMC7029862; doi:10.1371/journal.pone.0228731)
Supplement: Attachment — Submitted filename: Response to Reviewers.docx [file pone.0228731.s001.docx]

PLoS ONE	Brno, January 8, 2020

Editorial Office

Dear editor,

Please find attached our revised manuscript called

**Differences in right-to-left vs. left-to-right interventricular conduction times in patients indicated for cardiac resynchronization therapy**

by David Pospisil et al., **(**PONE-D-19-32599).

Thank you for your invitation to submit a revised version of the manuscript. We highly appreciate the opportunity to respond to reviewer’s suggestions on the paper to clarify all questions and to meet PLoS ONE’s publication criteria. We look forward to hearing from you regarding our submission. We would be glad to respond to any further questions and comments that you may have.

Best regards

On behalf of all the authors

Milan Sepsi, MD, PhD

Department of Internal Medicine and Cardiology

University Hospital Brno, Masaryk University, Jihlavska 20, 625 00 Brno, Czech Republic

Phone: +420 777 227 346, Fax: +420 53223 2611, E-mail: sepsi.milan@fnbrno.cz

**Response to Reviewer #1:**

Dear reviewer,

thank you for your valuable time and useful contribution. We highly appreciate your inputs you given to improve the manuscript. We hope you will find our explanations and manuscript edits enough to your convenience. A point-by-point reactions to your comments and critics are written below directly into received texts using blue colored italic font. Attached file “Revised Manuscript with Track Changes” contains marked revisions made in the manuscript.

Dear author,

the paper is well written, and the topic is very interesting. The protocol is clear and precise while results appear hard to understand after a single reading and should be improved considering that they seem to be a long list of figures and tables description.

*According to your proposition some corrections of the text have been applied. Nevertheless, the text of the “Results” section is quite short, substantial part of information is present in figures and their captions (which are inserted in the text of the manuscript immediately following the paragraph in which the figure is first cited).*

Moreover, an important limitation of this study is the small number of enrolled patients.

*We agree that the number is small and we have commented it in the limitations. Nevertheless, as a pilot study it can be sufficient to draw conclusions from a pathophysiological point of view. It is clear it deserves a further study on larger set of patients.*

At least, have you any data about response to resynchronization according to difference between inter-ventricular delay?

*Unfortunately, not yet. We are preparing a study to evaluate the phenomenon according to QRS width and responsiveness to CRT rigorously.*

Regarding this topic I would like to suggest this paper regarding QRS index that can be considered a surrogate of reduced interventricular conduction delay: Magnitude of QRS duration reduction after biventricular pacing identifies responders to cardiac resynchronization therapy.
Coppola G, Ciaramitaro G, Stabile G, DOnofrio A, Palmisano P, Carità P, Mascioli G, Pecora D, De Simone A, Marini M, Rapacciuolo A, Savarese G, Maglia G, Pepi P, Padeletti L, Pierantozzi A, Arena G, Giovannini T, Caico SI, Nugara C, Ajello L, Malacrida M, Corrado E.
Int J Cardiol. 2016 Oct 15;221:450-5.

*We find suggested paper very interesting regarding our topic and it has been added to the manuscript.*

**Response to Reviewer #2:**

Dear Dr. Mascioli,

thank you for your valuable time and useful contribution. We highly appreciate your inputs you given to improve the manuscript. We hope you will find our explanations and manuscript edits enough to your convenience. A point-by-point reactions to your comments and critics are written below directly into received texts using blue colored italic font. Attached file “Revised Manuscript with Track Changes” contains marked revisions made in the manuscript.

Reviewer #2: The paper from Sepsi and coll. is quite interesting, but it does present some methodological problems.

1. The position of the RV lead could have had an impact on results. It is not simply a matter of how many RV leads have been implanted in a group or in another, but that changing the RV lead position IN THAT patients could have lead to completely different results and this impacts negatively on the study itself.

*Of course, we considered that RV placement might have a significant impact to the interventricular conduction time and it obviously has. What we want to show is the fact that the differences of RV→LV and LV→RV conduction times are present regardless of RV lead placement in a given position.*

2. The definition itself of "septal RV lead" is quite generical: which part of the septum? Hissian, parahissian, septal RVOT? Again, this could have changed everything.

*The information on septal vs apical position is based on implanting physician’s declaration. Unfortunately more detailed information on the lead position is not available. The aim of our manuscript is introduction of the phenomenon. Relationships to particular positions would require its study on larger group of patients.*

3. The conclusions on correction on RV-LV lead delay (or viceversa; page 17, lines 213 - 214) and results of CRT is too simplicistic. FREEDOM Trial on use of QuickOpt did not demonstrate any benefit from this approach (it is almost the same concept, because the algorhitm measure the RV-LV delay during RV pacing and LV-RV delay during LV pacing and adjusts the VV interval so that the activation front is simultaneous)

*The text was modified.*

Furthermore, I do not understand (Abstract, page 8, line 30) what does that < 5 ms mean: could the Authors make it clearer or at least explain in the revision letter the meaning of this statement.

*In original text, there was a mistake – thank you: we considered interventricular conduction times difference lower than 5 milliseconds (ms) as a bidirectionally comparable conduction (because of measurement error), so. statement < 5 ms corrected to “>5 ms”.*

Finally, the whole manuscript needs a deep English language revision because some passages are unclear.

*A complete grammar, language and terminology check was done by scientific manuscript editing service to fit the US-English standards. We include PDF file with certification by Proof.Reading.Service.Com Ltd.*

**Response to Reviewer #3:**

Dear reviewer,

thank you for your valuable time and useful contribution. We highly appreciate your inputs you given to improve the manuscript. We hope you will find our explanations and manuscript edits enough to your convenience. A point-by-point reactions to your comments and critics are written below directly into received texts using blue colored font. Attached file “Revised Manuscript with Track Changes” contains marked revisions made in the manuscript.

Reviewer #3: Cardiac resynchronization therapy (CRT) is an established treatment for patients with heart failure (HF), impaired left ventricular (LV) function, and wide QRS complex.
Pospisil et al. described the phenomenon of interventricular conduction time differences and assessed relationship to various clinical and electrophysiological parameters. In fact the differences in conduction times from right ventricle to left ventricle and from left ventricle to right ventricle respectively were observed during biventricular devices implantation when changing pacing vector direction. In this study a pronounced differences (often up to 35 ms) were observed in substantial part of patients with dilatative cardiomyopathy (DCM), while this phenomenon was much less common in patients with coronary artery disease (CAD). The study is very interesting, presented in an appropriate fashion and are supported by interesting data. A limitation is represented by the small sample number (62 patients) to draw conclusions although the idea from a pathophysiological point of view is acceptable.

Moreover, it would be interesting to point out that other studies have also previously evaluated importance of CRT (see: “Non-responders to cardiac resynchronization therapy: Insights from multimodality imaging and electrocardiography. A brief review”, Doi: 10.1016/j.ijcard.2016.09.037).

This would certainly increase the article's quality. In conclusion, given the overall work, I accept manuscript with minor revision concerning a small implementation of bibliography, reporting the aforementioned work and other.

*We find the suggested paper very interesting regarding our topic and it has been added to the manuscript.*
